# Barriers to cancer pain management from the perspective of patients: A qualitative study

**DOI:** 10.1002/nop2.1093

**Published:** 2021-10-17

**Authors:** Samira Orujlu, Hadi Hassankhani, Azad Rahmani, Zohreh Sanaat, Abbas Dadashzadeh, Atefeh Allahbakhshian

**Affiliations:** ^1^ School of Nursing and Midwifery Tabriz University of Medical Sciences Tabriz Iran; ^2^ Emergency Medicine Research Team, Department of Medical Surgical Nursing School of Nursing and Midwifery Tabriz University of Medical Sciences Tabriz Iran; ^3^ Hematology and Oncology Research Center Tabriz University of Medical Sciences Tabriz Iran

**Keywords:** barriers, cancer, pain management, qualitative research

## Abstract

**Aim:**

The aim of this study was to explore the barriers to effective pain management in Iranian people with cancer.

**Design:**

A qualitative descriptive design was used.

**Methods:**

This qualitative descriptive study was performed on 14 people with cancer. Data were collected using semi‐structured interviews and analysed by Graneheim and Lundman's content analysis method.

**Results:**

Four main categories emerged in relation to barriers to pain management from the perspective of people with cancer. Categories included 1) accepting and enduring divine pain, 2) negative attitudes towards the effectiveness of analgesics, 3) patients’ low knowledge of pain self‐management methods and 4) neglected pain management. Barriers to pain management are multidimensional in nature consisting of patients, healthcare providers and system components. Therefore, attempts should be focused on the education of patients and healthcare providers about pain management and eliminating the shortcomings of the healthcare system.

## 1 INTRODUCTION

Pain is one of the most common symptoms in people with cancer, especially in the metastatic stage of the disease (Gress et al., 2020), which affects all aspects of a person's life (Haumann et al., 2017). Cancer pain reduces the quality of life by adverse impacts on emotional states, cognitive function, daily activities of life and relationship with family and other social networks (Gress et al., 2020; Russo & Sundaramurthi, 2019). It was reported that 51% of people with cancer experienced pain and 40% of those rated their pain moderate to severe (Van Den Beuken‐Van et al., 2016). According to the 2020 World Cancer Report, the numerous advances in cancer treatment are associated with increased survival in people with cancer and therefore the increased chronic pain of cancer in survivors (Wild CP, 2020).

Although cancer may be considered an end‐of‐life disease, the right to a healthy and pain‐free life should not be denied for people with cancer and every effort must be made to prevent their suffering (Wang et al., 2019). In other words, effective pain management could provide sufficient comfort and better function for each people with cancer (Nersesyan & Slavin, 2007). The care providers could use a variety of methods to reduce the pain of patients (Amenorpe, 2017). In 90% of people with cancer, pain can be relieved with effective pain management (Lou & Shang, 2017). Pain management is the reduction or control of pain by health professional services such as pain assessment, pain treatment, health education and psychological care (Lou & Shang, 2017). As the disease progresses and treatments become ineffective, pain management becomes the main goal, and in the final stages of the disease, it is the main focus of the care provided to the patient (Kelley et al., 2013).

### 1.1 Background

A study showed that pain is not treated in about one‐third of people with cancer in spite of the increase in awareness of cancer‐related pain in the literature (Haumann et al., 2017). Also despite advances in technology and the global use of potent opioids and other pain control drugs, 50%–80% of dying patients experience moderate to severe pain in their final weeks in hospital (Barkwell, 2005). Findings of a study in Iran also showed that even with the use of painkillers in people with cancer, they still suffer from pain and hence their pain should be managed properly (Salehifar et al., 2017).

Although cancer pain is a long‐standing problem (Erol et al., 2018), its effective management in people with cancer is still a major challenge for patients and their families and needs more attention and evaluation by the healthcare providers (Dabbous et al., 2017; Erol et al., 2018). Thinh et al. found that despite the availability of cancer pain management guidelines and effective analgesics, a significant number of people with cancer reported inadequate pain treatment which consequently affects their quality of life and sleep (Thinh et al., 2018). It is clear that healthcare providers need to be aware of barriers to pain management to plan effective educational programs and supportive services for improving the quality of life of patients and their families (Ovayolu et al., 2015).

Because all aspects of the pain experience, including pain perception, pain manifestation and pain treatment, occur in the socio‐cultural context, identifying barriers to pain management in this context is important for the effective treatment of cancer pain (Prastika et al., 2018). The objective of the present study was to explore the barriers to effective pain management in Iranian people with cancer. The findings of this study can provide in‐depth information for nurses and physicians on effective pain management of people with cancer.

## 2 METHODS

### 2.1 Design

The present research was a qualitative descriptive study conducted in 2020–2021 to explain the barriers to pain management in Iranian people with cancer. In this research approach, the phenomenon of interest is qualitatively described to acquire information about patient experiences and deal with important questions.

### 2.2 Setting and Participants

Participants were selected by purposive sampling method from patients referred to an oncology hospital, north‐western Iran. The hospital is the largest oncology centre in the region. A total of 14 patients aged 18 years and older were participated in the study after meeting inclusion criteria. All participants were informed of their diagnosis, experienced cancer‐related pain and undergoing chemotherapy, radiotherapy or surgery. They were able to understand and speak Persian or Turkish and expressed their informed and written consent. The samples were selected purposefully with maximum variety to provide a more comprehensive description of barriers to pain management. Sampling was continued until data saturation was reached. This means that in the interviews, new data were not provided by the participants. The demographic and disease characteristics of the participants are presented in Table 1.

**TABLE 1:** Demographic and disease characteristics of people with cancer

Characteristics	Mean± *SD* *N* (%)
Age, years	40.2 ± 8.7
Gender, female	8(57.2)
Working status	
*Housewife*	6(42.8)
*Working*	8(57.2)
Marital status, married	12(85.7)
Education level	
*Illiterate*	4(28.5)
*Primary school*	3(21.5)
*High school*	4(28.5)
*University*	3(21.5)
Diagnosis period, years	2.64 (±1.21)
Clinical diagnosis	
*Colon cancer*	2(14.3)
*Breast cancer*	3(21.4)
*Gastric cancer*	1(7.2)
*Lymphoma*	3(21.4)
*Lung cancer*	2(14.3)
*AML*	3(21.4)
Disease status, metastatic	11(78.6)
NRS	8.21(1.25)

### 2.3 Data collection

Data were collected by semi‐structured interviews which typically lasted 40–60 min. The interviews were conducted in a quiet room where the participants felt comfortable and their privacy was ensured. Participants were asked to describe their opinions, attitudes, beliefs and experiences about the factors that could impede effective cancer pain relief. The researcher began the interview with a broad question, followed by exploratory questions, and then, as the data collection and analysis progressed, more focus was placed on the questions, which were answered and determined by the participants’ answers.

The interviews were recorded by a digital tape recorder. This study was approved by the ethics committee (IR.TBZMED.REC.1399.147). Before each interview, the participants were given information about the objectives of the study, the method of interview, the right to participate in the study and the confidentiality of information. Informed consent was obtained from the participants before the interview.

### 2.4 Data analysis

Data analysis was performed simultaneously with data collection using qualitative content analysis by Graneheim and Lundman's method (Graneheim & Lundman, 2004). At the end of each session, the recorded interviews were transcribed verbatim and then analysed. In order to be immersed in the data, the text of each interview was read and reread multiple times and broken down into semantic units and then to the smallest meaningful units. The extracted codes were reviewed several times to be placed in categories and subcategories based on their semantic similarity. The first author (SO) coded and analysed the interviews with the second author (HH). Agreements were then reached with other authors. Also, the researcher tried to prevent her ideas and assumptions from being included in the data analysis. Data management and coding were performed with MAXQDA software.

### 2.5 Trustworthiness

During the study, rigor criteria for qualitative research (Guba & Lincoln, 1989) were used as follows: the constant engagement of the researcher with the research subject, participants and data; allocating enough time to conduct interviews; constantly reviewing and comparing codes in terms of similarities and differences; re‐checking the findings with participants; providing detailed data analysis and deep and rich descriptions of the research for readers; and using the corrective opinions of the research team members about the interview process, its analysis and the extracted data.

## 3 RESULTS

Fourteen people with cancer with a mean age of 40 years participated in this study. Eleven of those patients were in the metastatic phase of their disease. The mean pain intensity level of patients was 8.21 based on a numerical rating scale (NRS) (Table 1).

Table 1: Demographic and disease characteristics of people with cancer.

**FIGURE 1:**
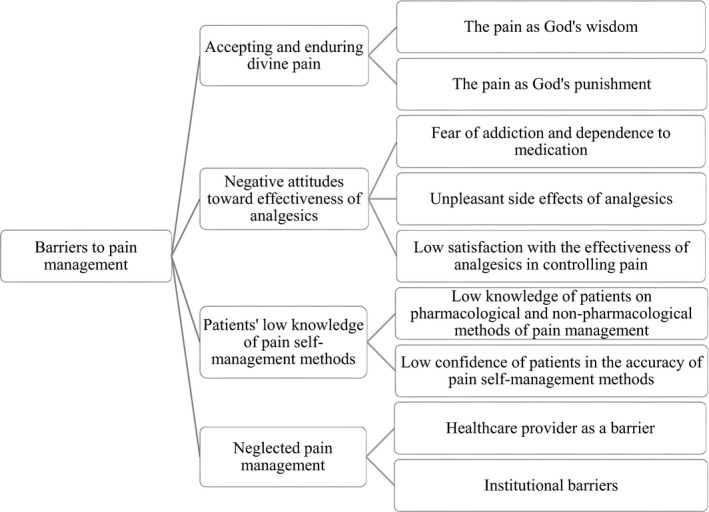
Barriers to cancer pain management from the perspective of patients

During the data analysis, four main categories emerged in relation to barriers to pain management from the perspective of people with cancer. Categories included accepting and enduring divine pain, negative attitudes towards effectiveness of analgesics, patients’ low knowledge of pain self‐management methods and neglected pain management. Each of these categories includes subcategories, which are summarized in Figure 1.

**Barrier** **1: Accepting and enduring divine pain**

The divine origin of pain was the meaning that patients attributed to the cancer pain. Religious beliefs such as the concept of God's will and interpretations of the causalities of health issues were dominant among patients. They believed that illness and pain were God's will and hence they cannot control it. According to this belief, the pain was an opportunity for patients to be cleansed of the sins committed in their lifetime. Such beliefs of patients about pain are the main barrier that leads to little or no motivation to implement pain management and hence to endure the pain. This category included two subcategories entitled the pain as God's wisdom and the pain as God's punishment.

### 3.1 The pain as God's wisdom

Since all participants in the study were Muslim, they described the pain in terms such as God's blessing, God's expediency and God's destiny. They believed that pain is one of the blessings of God.

"This pain is given by God and he considered pain expedient. It must be endured. The action of God cannot be stopped" (P1).

"I have been a believer for a long time. Even now, I call on God hundreds of times a day. I know that pain is the destiny of God. Even now, I rely only on God. I only hope God makes me better"(P10).

### 3.2 The pain as God's punishment

Some participants also considered pain as God's punishment and wrath and expressed it as atonement for sins. Hence, they believed that they should bear their pain and adopt Stoicism.

"I only ask God to heal me. I will get better only by the favor of God. I call on God every day. Only he can heal me and save me from this torment." (P6).

"I feel that God is angry with me. He wants to retaliate his anger on me and make me sick. Because we believe that sickness is the atonement for sins." (P4).

**Barrier** **2: Negative attitudes towards effectiveness of analgesics**

Participants cited fear of addiction and dependence on medication, unpleasant side effects of analgesics and low satisfaction with the effectiveness of analgesics in controlling pain as attitudinal barriers to cancer pain management.

### 3.3 Fear of addiction and dependence on medication

Some patients were concerned that relieving pain with medications would lead to long‐term addiction. They believed that pain medications, especially opioids, were dangerous and expressed their negative attitude towards medication. Therefore, they avoided consuming those medications until the pain became severe and unbearable.

"Sometimes I used opioids when my pain was so severe. It reduced my pain. But for fear of becoming addicted to these drugs and addiction, I did not want to take too much. I was very scared to get addicted." (P8).

### 3.4 Unpleasant side effects of analgesics

Patients talked about the side effects of analgesics such as nausea, constipation, loss of control and decreased level of consciousness which sometimes occurred with medications. They stated that they refused to take analgesics for fear of side effects.

"When I take analgesics, my stomach seems to swell and wants to explode. I have severe constipation that I prefer not to take analgesics." (P12).

"When I want to take analgesics, I get wet with sweat and feel bad. My head is confused and it seems that I am not in myself at all to the extent that I cannot pray and I have hallucinations. That's why I didn't use them anymore." (P7).

### 3.5 Low satisfaction with the effectiveness of analgesics in controlling pain

Participants reported that analgesics were less effective in achieving pain relief and they were less satisfied with the effects of prescribed analgesics. For this reason, they avoided taking these drugs.

"Analgesics have less effect. They are not more effective for pain. My pain still persists." (P5).

"I use different decoctions for my pain at home. Their effects are better than medications. I like the decoction very much." (P3).

**Barrier** **3: Patients’ low knowledge of pain self‐management methods**

Participants stated that they had little knowledge on the methods they could use at home to control their pain. They performed some methods for pain relief mainly based on trial and error, but not on scientific evidence.

### 3.6 Low knowledge of patients on pharmacological and non‐pharmacological methods of pain management

Our patients could not effectively control their pain and expressed their inability in reducing the pain. Insufficient knowledge on pharmacological and non‐pharmacological pain relief methods, such as the safe use of medications and distraction methods, was reported by most interviewees as a barrier to pain management. Patients stated that they did not receive any related training from physicians or nurses. Healthcare providers (HCPs) not only provided very little information to patients and their families but also spent very little time advising them.

"When I'm in pain, I do not know what to do. I cannot do anything. I wish my doctor and nurse would teach me a little bit about what to do when I am in pain." (P5).

"Many patients are not told what to do when they are in pain. I have not seen doctors give specialized training to reduce pain. Nurses do not mention this either. I can say that the medical staff talks about all the complications of cancer except pain." (P4).

### 3.7 Low confidence of patients in the accuracy of pain self‐management methods

The knowledge of our patients on pain self‐management methods was low, and they used all possible methods to relieve pain based on their previous experiences, guidance from others and unreliable sources. Patients reported the use of herbs, traditional and home remedies for pain control. However, patients were not sure about the effectiveness and accuracy of these methods and performed mostly based on trial and error. They reported that their knowledge of the adverse effects of alternative methods on the trend of their disease or drug interactions was low.

"I had a plant in the house. I got it from the herb shop. I do not know what its name is. My mother told me that in the past we used this plant. I pound this plant and heat it a little and put it on my chest. It made my pain a little less. I do not know whether it is effective or not." (P5).

"I tried many methods at home for pain relief. I really did not know what to do. I tried these myself." (P8).

**Barrier** **4: Neglected pain management**

Some participants agreed that their healthcare providers cannot properly manage their pain. They also identified some organizational factors as the most important barriers to effective pain management.

### 3.8 Healthcare provider as a barrier

Some patients reported HCPs as a barrier to effective pain management. Low prescription of analgesics and advising on pain tolerance, the lack of pain assessment by HCPs and the view that cancer equals pain were reported as significant barriers.

#### 3.8.1 Low prescription of analgesics and advising on pain tolerance

According to some patients, physicians refused to prescribe analgesics for their patients and are afraid of prescribing medications, especially opioids. They reported that doctors encouraged patients to endure the pain.

"My doctor did not prescribe much analgesics. He told me you have to endure the pain. When I told the doctor that I was in pain, he told me to endure the pain. Ever since I got this disease, I think there has not been a pain that I have not suffered." (P2).

"If my doctor prescribed a medicine for my pain, he would give me acetaminophen. I had to endure my pain myself. Because the doctor told me that analgesics are not good for you. Sometimes I had a lot of pain but I endure it." (P13).

"My doctor did not give me analgesics at all. I was constantly in pain. My doctor told me to endure the pain, but do not take analgesics." (P12).

#### 3.8.2 The lack of pain assessment by HCPs

Participants stated that their healthcare providers did not properly assess their pain. According to patients, physicians focus more on the disease and its treatment and less on the evaluation of the pain. Therefore, the lack of pain assessment could result in a misunderstanding of the patient's pain level that leads to poor pain management.

"I often tell my doctor that I am in pain. That is, despite the fact that the doctor knows his patient is in pain, he does not talk to the patient about the pain at all and does not ask about the level of pain." (P14).

"I have often seen staff say that this patient (me) is always in pain and exaggerates in expressing pain and has become addicted to drugs. Or they told that when this patient comes to the hospital, he says that I am in pain. The patient's perception of pain is different from the staff's perception of pain." (P11).

#### 3.8.3 The view that cancer equals pain

Findings from participants’ opinions showed that some physicians and nurses equated cancer with pain. They believed that pain is a part of cancer and did not help patients to relieve their pain. In such a view, even the prescription of analgesics is limited by physicians.

"Doctors say cancer is like that. Pain is a part of cancer. Maybe because they consider the pain as the nature of cancer, they often do not follow it at all." (P11).

Participant No. 2 recalled from his memory while hospitalized: "*A patient was moaning in the ward. A new registered nurse said to other nurses: "what is this case?" The nurses told her this case is a stomach cancer at end‐stage. She told, Ok, it is end‐stage! Why is she moaning? Nurses told her; doctors had told us the pain is a part of cancer."* That participant continued*:* "*Pain is accepted as a part of cancer. The pain has become so common for the staff that they equate cancer with pain. It is said that this is a normal process and should be painful*."

### 3.9 Institutional Barriers

Lack of sufficient analgesics in the wards and lack of patient referral system were reported by patients as the barriers to their pain management.

#### 3.9.1 Lack of sufficient analgesics in the wards

The findings showed that there were not enough analgesics, especially opioids, in the hospital wards and the patients waited a long time to receive medications. Therefore, patients had to endure severe pain until the drug reaches the ward.

"I have been in pain for several hours. They say the medicine is coming now, it is coming now. I am in a lot of pain. Please order me to inject opioid as soon as possible. I only live with the opioid. "(P13).

#### 3.9.2 Lack of patient referral system

According to findings, some patients complained about the lack of inter‐disciplinary collaboration between physicians and pain specialists. Participants stated that physicians did not refer them to clinics running by pain specialists. Many patients were even unaware of the existence of such clinics.

"I have not seen doctors refer patients to pain clinics. The rate of referrals is rare and close to zero. I have not seen any collaboration between our doctors and pain specialist. Referral of patients to the pain clinic is very rare." (P2).

"I used to take tramadol but the doctor stopped it and told me it was addictive. I myself got familiar with the pain clinic by a relative and went there. But the doctor at the pain clinic prescribed tramadol again." (P9).

## 4 DISCUSSION

The overall aim of this study was to discover the barriers to pain management in people with cancer in Iran. Findings showed that acceptance and tolerance of divine pain, negative attitude towards the effectiveness of analgesics, low knowledge of patients about pain self‐management methods and neglected pain management can affect pain management in this population.

Accepting and enduring divine pain was the dominant religious belief of patients about the pain of cancer, with two subcategories of pain as God's wisdom and pain as God's punishment. The interviews showed that patients believed in God's pre‐determination and attributed the pain to God's will. In this view, pain provides an opportunity to purify sins and receive greater rewards in the afterlife. Due to religious beliefs, the majority of patients would prefer to endure pain rather than to express it resulting in less motivation to follow medical advice. Adopting such a view of pain also prevents patients from expressing the pain and its effective treatment. In line with our results, Al‐Ghabeesh et al. showed that people with cancer believed that they were not able to influence their disease in any way because of fatalistic beliefs (Al‐Ghabeesh et al., 2020). Gerber et al. also noted that some patients and families were refused or denied pain relief because of their religious, cultural or personal beliefs (Gerber et al., 2021). Cultural differences were also reported as barriers to pain management by Gunnarsdottir et al. (Gunnarsdottir et al., 2017). The findings of the present study showed how the cultural context and the way of interpretation of pain by patients influenced their pain management. Therefore, the awareness of HCPs about the impact of patients’ religious and cultural beliefs on their desire to receive palliative care influences pain management.

Negative attitudes towards the effectiveness of analgesics such as opioids were among the barriers highlighted by participants. The findings of this study showed that patients were concerned about opioid addiction and its side effects. The fear of addiction was listed as one of the most important barriers to pain management among Jordanian patients with cancer (Al‐Ghabeesh et al., 2020). Yates et al. also found that the majority of patients stated addiction to medication as a barrier and believed in a real risk of addiction (Yates et al., 2002). Patients in the study of Mercadante et al. identified constipation, nausea, vomiting and a feeling of tiredness and lethargy as worrying side effects of analgesics and avoided taking the medication (Mercadante et al., 2021). Opioids side effects and fear of addiction have also highlighted as attitudinal barriers by participants of the previous studies (Konstantis & Exiara, 2018; Kwon, 2014). Participants in the study of Konstantis also cited low satisfaction with the effectiveness of analgesics in controlling pain as a factor in avoiding analgesics (Konstantis & Exiara, 2018; Michaelson Monaghan, 2019). The less effectiveness of opioids on pain control was reported as an attitudinal barrier by Yates et al. (Yates et al., 2002). Patients who have attitudinal barriers to pharmacological pain management use fewer analgesics than those who do not have. Consequently, these patients experience more pain, more pain‐related interference in daily activities and more decreased quality of life (Gunnarsdottir et al., 2017). Suffering from severe pain and prolonged pain tolerance because of the negative attitude towards medication seems irrational belief that imposes irreversible consequences for patients’ health. Patients’ attitudes can be improved by providing training on addiction prevention, side effects, timing and dosage of analgesics. In a study, pain education for patients reduced the pain levels, increased treatment satisfaction and prevented patient barriers to pain management (Uysal, 2018). Therefore, oncology nurses should play an active role in educating patients and their families about pain and its treatment.

Poor knowledge on pharmacological and non‐pharmacological methods of pain management and low confidence in using these methods were serious barriers that participants expressed. There was considerable agreement among the participants on this issue. Analysis of interview data showed that patients have poor knowledge and information about their disease and management of its symptoms, especially pain. The findings showed that patients were unaware of non‐pharmacological treatments such as relaxation and mind distraction techniques, and cognitive‐behavioural interventions. Patients stated that they, unfortunately, did not receive any training on non‐pharmacological treatments from HPCs and therefore preferred to seek required information from alternative sources such as the Internet or relatives. This could result in the improper use of various pharmacological and non‐pharmacological methods for controlling the pain. On the other hand, according to patients, they are not sure about the accuracy of these methods and perform them based on trial and error. It means that there is a knowledge gap between HCPs and patients. It is clearly right to say that the negative attitude towards analgesics is caused by the lack of knowledge in patients. Therefore, it needs a better interaction between HCPs and patients to provide proper information to them. Increasing knowledge about pain could establish a basis for forming a better attitude towards pain management in people with cancer (Lou & Shang, 2017). Most participants in the study of Al‐Ghabeesh et al. also reported a deficit of knowledge on cancer care and pain management due to the lack of communication with HCPs (Al‐Ghabeesh et al., 2020). Charalambous et al. found inadequate patient education as a threat to patient safety, i.e. the use of opioid drugs by patients only based on their limited information is a threat to their safety (Charalambous et al., 2019). Poor knowledge of HCPs and patients on the application of pain relief strategies were reported as serious barriers in reducing pain (Nayak et al., 2018). A comprehensive review found that patient education programs could help to correct the misconceptions and hence reduce cancer pain slightly (Gress et al., 2020). Similarly, a booklet was effective in changing misconceptions about opioids (Gulati, 2021). Therefore, in the light of effective communication, nurses should be aware of all pain control methods used by patients to promote positive pain‐relieving behaviours and coping skills in patients.

Neglected pain management was another important category that was extracted from the interviews with participants. From the participants’ point of view, HPCs were a barrier to pain management in people with cancer. Participants stated that some physicians were refuse to prescribe analgesics and opioids and advised patients to tolerate pain. Consistent with this finding, Onsongo showed that some physicians did not like prescribing analgesics and ignored reports of patients’ pain (Onsongo, 2020). According to Gerber et al., nurses reported the refusal of some physicians to prescribe pain medication (Gerber et al., 2021). It is also reported that physicians fear the responsibility of prescribing opioid analgesics such as morphine. The main reasons for the refusal of physicians could be the lack of awareness of the side effects of opioids or having misconceptions about these drugs (Charalambous et al., 2019). Lack of experience and knowledge of physicians and nurses about pain treatment, the basic principles of pain control, side effects control, addiction and drug dosage could contribute to the avoidance of opioids and analgesics prescription (Uysal, 2018). Therefore, HCPs should be trained on pain management and treatment using opioids to increase the effects and the safe use of these medications.

The findings of the present study indicated that the pain of people with cancer was not evaluated by HCPs. Physicians and nurses did not use standard tools for pain assessment and had a general judgment on the level of pain. This could lead to the misunderstanding of the patient's pain level and thus insufficient pain relief. Pain assessment can form the basis of successful pain management (Gulati, 2021). The poor assessment of pain and refusal of physicians to prescribe opioids were reported as major challenges in managing cancer symptoms (Gerber et al., 2021; Kwon, 2014). Therefore, physicians and nurses should be trained on reliable pain assessment tools to kindly assess the pain of patients in an empathetic relationship.

Some HPCs believed that cancer equals pain and is a part of cancer. Such a view of physicians made them less sensitive to relieving patients’ pain. It is shown that poor knowledge, insufficient experience and common misconceptions of physicians and nurses about cancer pain are among the most significant challenges (Gulati, 2021). In contrast, a Chinese study showed that most physicians give almost equal priority to the treatment of cancer pain and cancer itself (Zhang et al., 2015). Therefore, improving the knowledge and beliefs of the health profession scheduled right from the start of the university course could provide a basis for effective pain management (Gulati, 2021). The present study also emphasized the training programs for clinicians to provide detailed information on the nature of pain and to remember the need for assessing pain in people with cancer.

The lack of sufficient opioid analgesics in the wards and patient referral system were the institutional barriers reported by the participants. Due to the lack of sufficient opioids, patients waited a long time to receive drugs and had to endure the pain. |Similarly, the lack of access to a wide range of analgesics and reduced access to essential drugs were among the barriers reported in the previous studies (Bruera & Paice, 2015; Kwon, 2014). Access to the cancer care centres because of their geographical location and health insurance were reported by people with cancer as their main concerns. They also identified limited access to medications and other support services as barriers to pain management (Gerber et al., 2021). In developed countries, physicians have access to a wide range of opioids, while in the developed countries there are some limitations (Kwon, 2014). In Iran, as a developing country, pain management of people with cancer could be improved by providing easy access to a variety of health services.

The lack of multidisciplinary collaboration between physicians and pain specialists was distinguished as a remarkable gap by our patients. Most of the patients were unaware of the existence of pain‐relieving specialties and centres that provide professional services. In line with our findings, Kwon also identified institutional barriers as lack of collaboration between multiple healthcare providers, difficulty in accessing pain intervention services, lack of support from pain specialists and palliative care (Kwon, 2014). Improving collaboration between physicians and pain specialists could improve pain management in people with cancer (Gulati, 2021). A survey showed that most physicians do not refer patients to pain or palliative care specialists because of difficulty in accessing or scheduling appointments with these services (Kwon, 2014). With medical collaborative services, the pain of patients could be controlled in a shorter time and in a more effective way.

### 4.1 Limitations

This study was performed during the COVID‐19 pandemic. Since people with cancer need more care against coronavirus, the researcher had difficulties entering the oncology wards, having access to a variety of participants and extending the interview time. These limitations may affect the results of this study.

### 4.2 Implications for practice

Patients’ religious beliefs about pain and the consequent negative impact on their pain management should be understood by medical staff, especially nurses. Nurses can educate people with cancer to increase the knowledge of patients and change their negative attitudes towards accurate pain management. It is necessary to train physicians and nurses to change their attitudes and correct their misconceptions about the nature of patient's pain. They are advised to use standard tools to assess the pain of patients regularly. Physicians and nurses could provide consultation for people with cancer about the palliative care services.

## 5 CONCLUSION

This qualitative study explored the experiences of people with cancer about the various barriers to pain management. According to the findings, barriers to pain management were explored as multidimensional in nature comprising factors related to patients, healthcare providers and the system. Therefore, effective pain management requires a multidimensional approach and interdisciplinary interventions by collaborative teams. The religious beliefs of patients, their poor knowledge and negative attitudes towards controlling and managing cancer pain were identified as personnel barriers to pain management. The low willingness of physicians to prescribe analgesics and improper assessment of patients’ pain were barriers associated with healthcare providers that hampered the successful management of patient's pain. Patients also complained about the lack of opioid analgesics in the wards and the lack of patient referral systems as institutional barriers.

## CONFLICTS OF INTEREST

All authors have no conflicts of interest.

## AUTHOR CONTRIBUTIONS

Samira Orujlu and Hadi Hassankhani designed and conducted the study and prepared the manuscript. Samira Orujlu, Hadi Hassankhani, Azad Rahmani, Abbas Dadashzadeh and Atefeh Allahbakhshian performed data analysis. Zohreh Sanaat helped to select the study participants. Versions of the manuscripts were shared, revised and written by all authors. All authors have read and approved the submitted manuscript.

## Data Availability

Data are openly available on request from the authors.

## References

[nop21093-bib-0002] Al‐Ghabeesh, S. H. , Bashayreh, I. H. , Saifan, A. R. , Rayan, A. , & Alshraifeen, A. A. (2020). Barriers to effective pain management in cancer patients from the perspective of patients and family caregivers: A qualitative study. Pain Management Nursing, 21(3), 238–244. 10.1016/j.pmn 31494027

[nop21093-bib-0003] Amenorpe, F. D. (2017). Chronic pain experiences among advanced cancer patients in the accra metropolis. [Master of philosophy nursing degree, University of Ghana], http://ugspace.ug.edu.gh/handle/123456789/23209

[nop21093-bib-0004] Barkwell, D. (2005). Cancer pain: Voices of the Ojibway people. Journal of Pain and Symptom Management, 30(5), 454–464. 10.1016/j.jpainsymman 16310619

[nop21093-bib-0031] Beuken‐Van, M. H. , Hochstenbach, L. M. , Joosten, E. A. , Tjan‐Heijnen, V. C. , & Janssen, D. J. (2016). Update on prevalence of pain in patients with cancer: systematic review and meta‐analysis. Journal of Pain and Symptom Management, 51(6), 1070‐1090. 10.1016/j.jpainsymman.2015.12.340 27112310

[nop21093-bib-0005] Bruera, E. , & Paice, J. A. (2015). Cancer pain management: Safe and effective use of opioids. American Society of Clinical Oncology Educational Book, 35(1), e593–e599. 10.14694/EdBook_AM.2015.35.e593 25993228

[nop21093-bib-0006] Charalambous, A. , Zorpas, M. , Cloconi, C. , & Kading, Y. (2019). Healthcare professionals’ perceptions on the use of opioid analgesics for the treatment of cancer‐related pain in Cyprus: A mixed‐method study. SAGE Open Medicine, 7, 2050312119841823. 10.1177/2050312119841823 31057793 PMC6452428

[nop21093-bib-0007] Dabbous, M. K. , Sakr, F. R. , Bouraad, E. P. , Safwan, J. H. , Akel, M. G. , & Cherfan, M. M. (2017). Pain assessment and management in cancer patients. Journal of Health Research and Reviews in Developing Countries, 4(1), 8. 10.4103/2394-2010.199324

[nop21093-bib-0008] Erol, O. , Unsar, S. , Yacan, L. , Pelin, M. , Kurt, S. , & Erdogan, B. (2018). Pain experiences of patients with advanced cancer: A qualitative descriptive study. European Journal of Oncology Nursing, 33, 28–34. 10.1016/j.ejon 29551174

[nop21093-bib-0009] Gerber, K. , Willmott, L. , White, B. , Yates, P. , Mitchell, G. , Currow, D. C. , & Piper, D. (2021). Barriers to adequate pain and symptom relief at the end of life: A qualitative study capturing nurses’ perspectives. Collegian, 28, 606–613. 10.1016/j.colegn.2021.02.008

[nop21093-bib-0010] Graneheim, U. H. , & Lundman, B. (2004). Qualitative content analysis in nursing research: Concepts, procedures and measures to achieve trustworthiness. Nurse Education Today, 24(2), 105–112. 10.1016/j.nedt.2003.10.001 14769454

[nop21093-bib-0011] Gress, K. L. , Charipova, K. , Kaye, A. D. , Viswanath, O. , & Urits, I. (2020). An Overview of Current Recommendations and Options for the Management of Cancer Pain: A Comprehensive Review. Oncology and Therapy, 8(2), 251–259. 10.1007/s40487-020-00128-y 32894414 PMC7683745

[nop21093-bib-0012] Guba, E. G. , & Lincoln, Y. S. (1989). Fourth generation evaluation. SAGE.

[nop21093-bib-0013] Gulati, R. R. (2021). The Challenge of Cancer Pain Assessment. The Ulster Medical Journal, 90(1), 37. 33642634 PMC7907907

[nop21093-bib-0014] Gunnarsdottir, S. , Sigurdardottir, V. , Kloke, M. , Radbruch, L. , Sabatowski, R. , Kaasa, S. , & Klepstad, P. (2017). A multicenter study of attitudinal barriers to cancer pain management. Supportive Care in Cancer, 25(11), 3595–3602. 10.1007/s00520-017-3791-8 28653107

[nop21093-bib-0015] Haumann, J. , Joosten, E. B. A. , & van den Beuken‐van, M. H. (2017). Pain prevalence in cancer patients: Status quo or opportunities for improvement? Current Opinion in Supportive and Palliative Care, 11(2), 99–104. 10.1097/SPC.0000000000000261 28306569

[nop21093-bib-0016] Kelley, M. , Demiris, G. , Nguyen, H. , Oliver, D. P. , & Wittenberg‐Lyles, E. (2013). Informal hospice caregiver pain management concerns: A qualitative study. Palliative Medicine, 27(7), 673–682. 10.1177/0269216313483660 23612959 PMC3950803

[nop21093-bib-0017] Konstantis, A. , & Exiara, T. (2018). Family caregiver beliefs and barriers to effective pain management of cancer patients in home care settings. *Journal of B.U.ON*.*: O* *fficial* *journal of the Balkan Union of* . Oncology, 23(7), 144–152. 30722124

[nop21093-bib-0018] Kwon, J. H. (2014). Overcoming barriers in cancer pain management. Journal of Clinical Oncology, 32(16), 1727–1733. 10.1200/JCO.2013.52.4827 24799490

[nop21093-bib-0019] Lou, F. , & Shang, S. (2017). Attitudes towards pain management in hospitalized cancer patients and their influencing factors. Chinese Journal of Cancer Research, 29(1), 75. 10.21147/j.issn.1000-9604 28373756 PMC5348478

[nop21093-bib-0020] Mercadante, S. , Adile, C. , Tirelli, W. , Ferrera, P. , Penco, I. , & Casuccio, A. (2021). Barriers and Adherence to Pain Management in Advanced Cancer Patients. Pain Practice, 21(4), 388–393. 10.1111/papr.12965 33200548

[nop21093-bib-0021] Michaelson Monaghan, E. (2019). Pain assessment tools for malingering in patients with chronic pain. Plus: Differentiating between this exaggeration and factitious disorder, somatization, and pain catastrophizing. Practical Pain Management, 19(4), 23–25.

[nop21093-bib-0022] Nayak, M. G. , George, A. , & Vidyasagar, M. (2018). Perceived barriers to symptoms management among family caregivers of cancer patients. Indian Journal of Palliative Care, 24(2), 202. 10.4103/IJPC.IJPC-27-18 29736126 PMC5915890

[nop21093-bib-0023] Nersesyan, H. , & Slavin, K. V. (2007). Current approach to cancer pain management: Availability and implications of different treatment options. Therapeutics and Clinical Risk Management, 3(3), 381. 18488078 PMC2386360

[nop21093-bib-0024] Onsongo, L. N. (2020). Barriers to Cancer Pain Management Among Nurses in Kenya: A Focused Ethnography. Pain Management Nursing, 21(3), 283–289. 10.1016/j.pmn 31561974

[nop21093-bib-0025] Ovayolu, Ö. , Ovayolu, N. , Aytaç, S. , Serçe, S. , & Sevinc, A. (2015). Pain in cancer patients: Pain assessment by patients and family caregivers and problems experienced by caregivers. Supportive Care in Cancer, 23(7), 1857–1864. 10.1007/s00520-014-2540-5 25471183

[nop21093-bib-0026] Prastika, D. , Kitrungrote, L. , & Damkliang, J. (2018). Pain‐management strategies among hospitalized trauma patients: A preliminary study in a teaching hospital in Indonesia. Enfermeria Clinica, 28, 158–161. 10.1016/S1130-8621(18)30058-5

[nop21093-bib-0027] Russo, M. M. , & Sundaramurthi, T. (2019). An overview of cancer pain: Epidemiology and pathophysiology. Seminars in Oncology Nursing, 35(3), 223–228. 10.1016/j.soncn 31085106

[nop21093-bib-0028] Salehifar, E. , Hazeghpasand, R. , Keyhanian, S. , Ala, S. , & Ahangar, N. (2017). Evaluating Pain Management among Cancer Patients in a Chemotherapy Center. Journal of Mazandaran University of Medical Sciences, 27(150), 89‐97.

[nop21093-bib-0029] Thinh, D. H. Q. , Sriraj, W. , Mansor, M. , Tan, K. H. , Irawan, C. , Kurnianda, J. , Nguyen, Y. P. , Ong‐Cornel, A. , Hadjiat, Y. , Moon, H. , & Javier, F. O. (2018). Patient and physician satisfaction with analgesic treatment: Findings from the analgesic treatment for cancer pain in Southeast Asia (ACE) study. Pain Research and Management, 2018, 2193710. 10.1155/2018/2193710 29849841 PMC5932441

[nop21093-bib-0030] Uysal, N. (2018). Clearing Barriers in Cancer Pain Management: Roles of Nurses. International Journal of Caring Sciences, 11(2), 1323–1327.

[nop21093-bib-0032] Wang, N. , Dong, Y. , Zhao, L. , Zhao, H. , Li, W. , & Cui, J. (2019). Factors associated with optimal pain management in advanced cancer patients. Current Problems in Cancer, 43(1), 77–85. 10.1016/j.currproblcancer 29934220

[nop21093-bib-0001] Wild, C. P. , Weiderpass, E. , & Stewart, B. W. , editors (2020). World Cancer Report: Cancer Research for Cancer Prevention. International Agency for Research on Cancer. Available from: http://publications.iarc.fr/586. Licence: CC BY‐NC‐ND 3.0 IGO 39432694

[nop21093-bib-0033] Yates, P. M. , Edwards, H. E. , Nash, R. E. , Walsh, A. M. , Fentiman, B. J. , Skerman, H. M. , & Najman, J. M. (2002). Barriers to effective cancer pain management: A survey of hospitalized cancer patients in Australia. Journal of Pain and Symptom Management, 23(5), 393–405. 10.1016/s0885-3924(02)00387-1 12007757

[nop21093-bib-0034] Zhang, Q. , Yu, C. , Feng, S. , Yao, W. , Shi, H. , Zhao, Y. , & Wang, Y. (2015). Physicians' Practice, Attitudes Toward, and Knowledge of Cancer Pain Management in China. Pain Medicine, 16(11), 2195–2203. 10.1111/pme.12819 26118400

